# Non-blocking anti-PD-L1 nanobody conjugated to TLR7 agonist mediates macrophage/NK cell-associated antitumor effects

**DOI:** 10.1016/j.apsb.2025.05.005

**Published:** 2025-05-16

**Authors:** Chao Hu, Chen Chen, Xiaolu Yu, Zhiying Li, Feng Tang, Qi Sun, Yiru Long, Likun Gong

**Affiliations:** a State Key Laboratory of Drug Research, Shanghai Institute of Materia Medica, Chinese Academy of Sciences, Shanghai 201203, China; b University of Chinese Academy of Sciences, Beijing 100049, China; c School of Public Health, China Medical University, Shenyang 110122, China; d School of Chinese Materia Medica, Nanjing University of Chinese Medicine, Nanjing 210023, China; e Zhongshan Institute for Drug Discovery, Shanghai Institute of Materia Medica, Chinese Academy of Sciences, Zhongshan 528400, China

To the Editor,

Antibody-drug conjugates (ADCs) have garnered significant attention in cancer therapy and are progressively being adopted in clinical applications^1^^,^^2^. Immune-stimulating antibody conjugates (ISAC) represent an innovative category of ADCs that link pattern recognition receptor (PRR) agonists to antibodies, aiming to impede tumor progression by eliciting the anti-tumor immune response^3^^,^^4^. By substituting small molecule toxins that directly kill tumor cells, ISAC can be broadened to encompass a greater variety of tumor-associated antigens (TAA) and has demonstrated remarkable efficacy in preclinical models. Nonetheless, constrained by a narrow therapeutic window, ISAC has encountered challenges in clinical trials, necessitating the urgent exploration of more suitable combinations of TAA targets and PRR agonists for safer and more efficacious drug design^4^^,^^5^. And an in-depth comprehension of the mechanisms by which antibodies and PRR agonists contribute to ISAC efficacy will enhance ISAC optimization. Our previous study preliminarily explored the potent antitumor efficacy of a programmed cell death 1 ligand 1 (PD-L1) antibody coupled with a Toll-like receptor (TLR) 7 agonist^6^. In this work, we aimed to evaluate in depth the roles that PD-L1 antibody and TLR7 agonist play in ISAC, as well as the effectiveness and mode of action of a novel PD-L1/TLR7-targeted ISAC.

## 1 Non-blocking anti-PD-L1 nanobody mediates endocytosis of PD-L1

**Figure 1:**
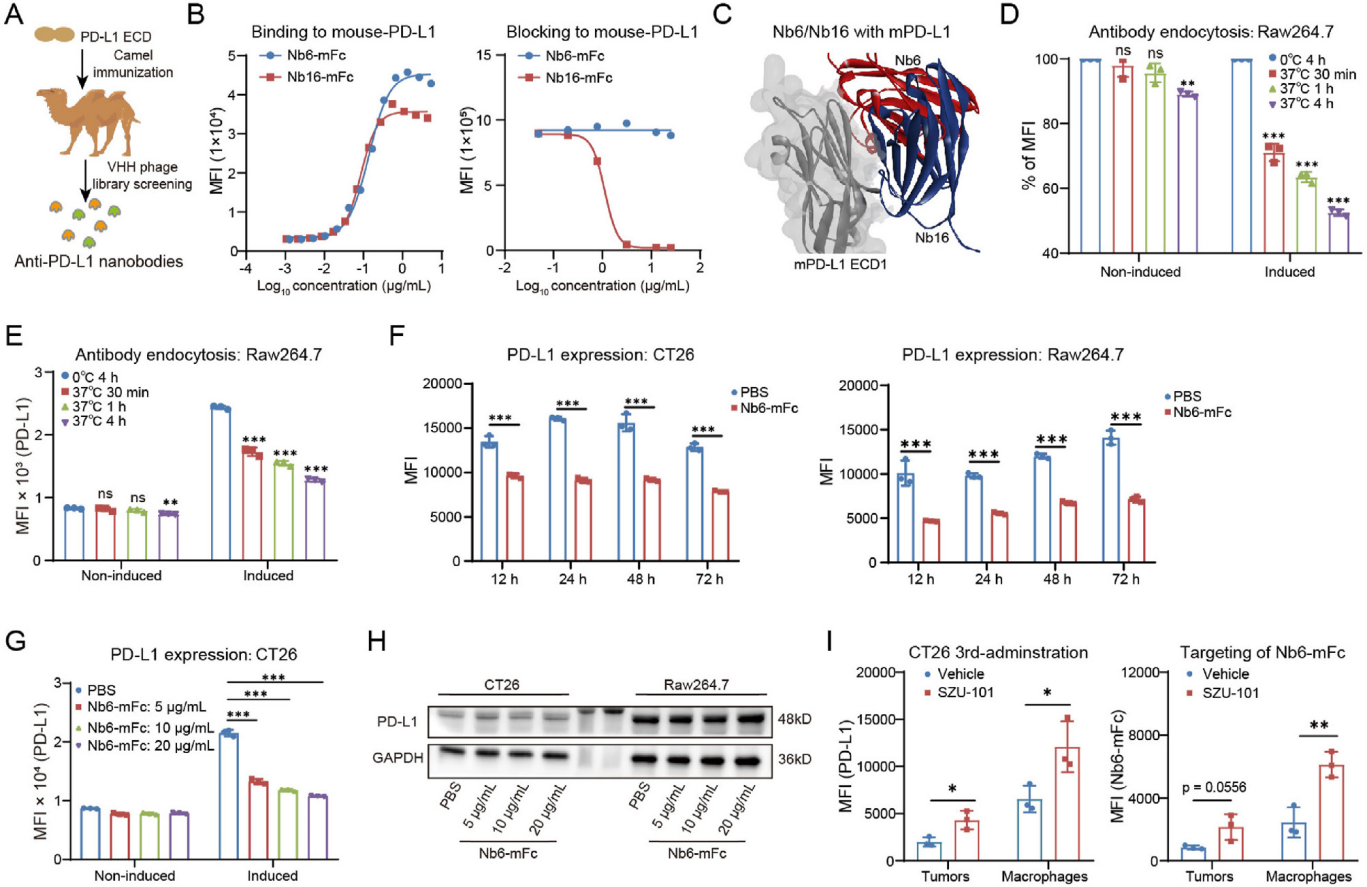
Characterization of non-blocking anti-PD-L1 nanobody Nb6. (A) Camel immunization and anti-PD-L1 nanobodies screening. (B) The binding and blocking activity of Nb6-mFc was tested by flow cytometry. (C) Complex structures of Nb6/PD-L1 and Nb16/PD-L1 predicted by AlphaFold. (D, E) Endocytosis analysis of Nb6-mFc in Raw264.7 cells induced by IFN-*γ* for PD-L1 expression or not (*n* = 3). (F) Effect of 5 μg/mL Nb6-mFc treatment for different times on membrane PD-L1 in CT26 cells, Raw264.7 cells (*n* = 3). (G) Effect of different concentrations of Nb6-mFc on membrane PD-L1 in CT26 cells after 12 h (*n* = 3). (H) Changes in total PD-L1 protein in CT26 cells and Raw264.7 cells after 12 h of treatment with different concentrations of Nb6-mFc. (I) Left, PD-L1 expression in tumor cells and macrophages after 3 doses of SZU-101 peritumoral administrations (*n* = 3). Right, targeting of Nb6-mFc to intratumoral tumor cells and macrophages after SZU-101 administration (*n* = 3). The data are presented as mean ± SEM. ∗*P* < 0.05; ∗∗*P* < 0.01; ∗∗∗*P* < 0.001; ns not significant by unpaired *t*-test or one-way/two-way ANOVA followed by Tukey's multiple comparisons test.

In order to assess the necessity of PD-1/PD-L1 blocking activity, we sought to acquire a non-blocking PD-L1 antibody to investigate the role of PD-L1 antibodies in ISAC. After camel immunization with the PD-L1 extracellular domain (ECD), a novel PD-L1 nanobody named Nb6 was obtained by phage display techniques (Fig. 1A). Using HEK293T-mPD-L1 cell-based flow cytometry (Fig. 1B), compared to the control nanobody Nb16, we determined that Nb6 failed to disrupt the mouse PD-1/PD-L1 interaction but could bind to mouse PD-L1 with a high affinity (EC_50_ value of 1.72 nmol/L). Nb6 is a novel non-blocking PD-L1 nanobody. In addition, we predicted the structures of the complexes of Nb6/PD-L1 and Nb16/PD-L1 by Alphafold2-multimer^7^, and found that there are indeed significant spatial differences between Nb6 and Nb16 for the epitopes on PD-L1 (Fig. 1C and Supporting Information Fig. S1).

Despite Nb6 lacking blocking efficacy, we questioned if this antibody exhibited effective endocytosis. We detected the internalization of Nb6-mFc in the murine monocyte/macrophage cell line Raw264.7, mouse peritoneal macrophages (PM), mouse CT26 tumor cells, and mouse B16–F10 tumor cells (Fig. 1D and E, and Supporting Information Fig. S2). Interferon-*γ* (IFN-*γ*) was used to induce upregulation of PD-L1 expression in these cells. The results indicated that Nb6-mFc was internalized by PD-L1-expressing immune cells and tumor cells, with the endocytosis rate positively associated to PD-L1 expression levels. The endocytosis rate of Nb6-mFc in macrophages was much higher than in tumor cells. Nb6-mFc demonstrated an endocytosis rate of up to 47.51% after 4 h in IFN-*γ*-induced Raw264.7 cells (Fig. 1D).

Internalization of Nb6-mFc may promote PD-L1 endocytosis. We verified the lack of epitope competition between Atezolizumab (Tecentriq) and Nb6, which is applicable for detecting membrane PD-L1 following Nb6 endocytosis (Supporting Information Fig. S3). Membrane PD-L1 levels in CT26 cells and Raw264.7 cells remained low for at least 72 h accompanying Nb6-mFc treatment (Fig. 1F). And we treated CT26 cells with varying concentrations of Nb6-mFc and found that a low concentration of Nb6-mFc alone markedly reduced membrane PD-L1 (Fig. 1G). Subsequently, we intended to investigate if PD-L1-dependent endocytosis of Nb6 influences PD-L1 stability. Our findings indicate that total PD-L1 levels remained unchanged in the cells, implying that the Nb6/PD-L1 complex was not subjected to degradation following endocytosis (Fig. 1H). The aforementioned results demonstrate that Nb6 is a non-blocking PD-L1 nanobody exhibiting PD-L1-dependent endocytosis activity, suitable for the development of innovative ISACs.

Recent studies show that macrophages inside the tumor microenvironment express elevated levels of PD-L1 compared to tumor cells^8^. Given that Nb6 was internalized more by macrophages than by tumor cells, we proceeded to assess the impact of the TLR7 agonist SZU-101 on PD-L1 expression in macrophages. SZU-101, as a potent TLR7 small molecule agonist that can initiate an innate immune response, was used as an antitumor drug candidate and vaccine adjuvant in our previous work^6^^,^^9^. SZU-101 significantly promoted PD-L1 expression by Raw264.7 cells and PM *in vitro* (Supporting Information Fig. S4). *In vivo*, peritumoral injection of SZU-101 markedly increased PD-L1 expression in tumor cells and intratumoral macrophages, particularly in macrophages (Fig. 1I). Therefore, we hypothesized that TLR7 agonist administration could promote anti-PD-L1 antibody targeting to intratumoral macrophages. We found that SZU-101 administration biased the targeting of Nb6-mFc to intratumoral macrophages, with Nb6-mFc binding increased to 2.5-fold (Fig. 1I).

## 2 Non-blocking anti-PD-L1 nanobody conjugated to TLR7 agonist exerts potent anti-tumor efficacy

**Figure 2:**
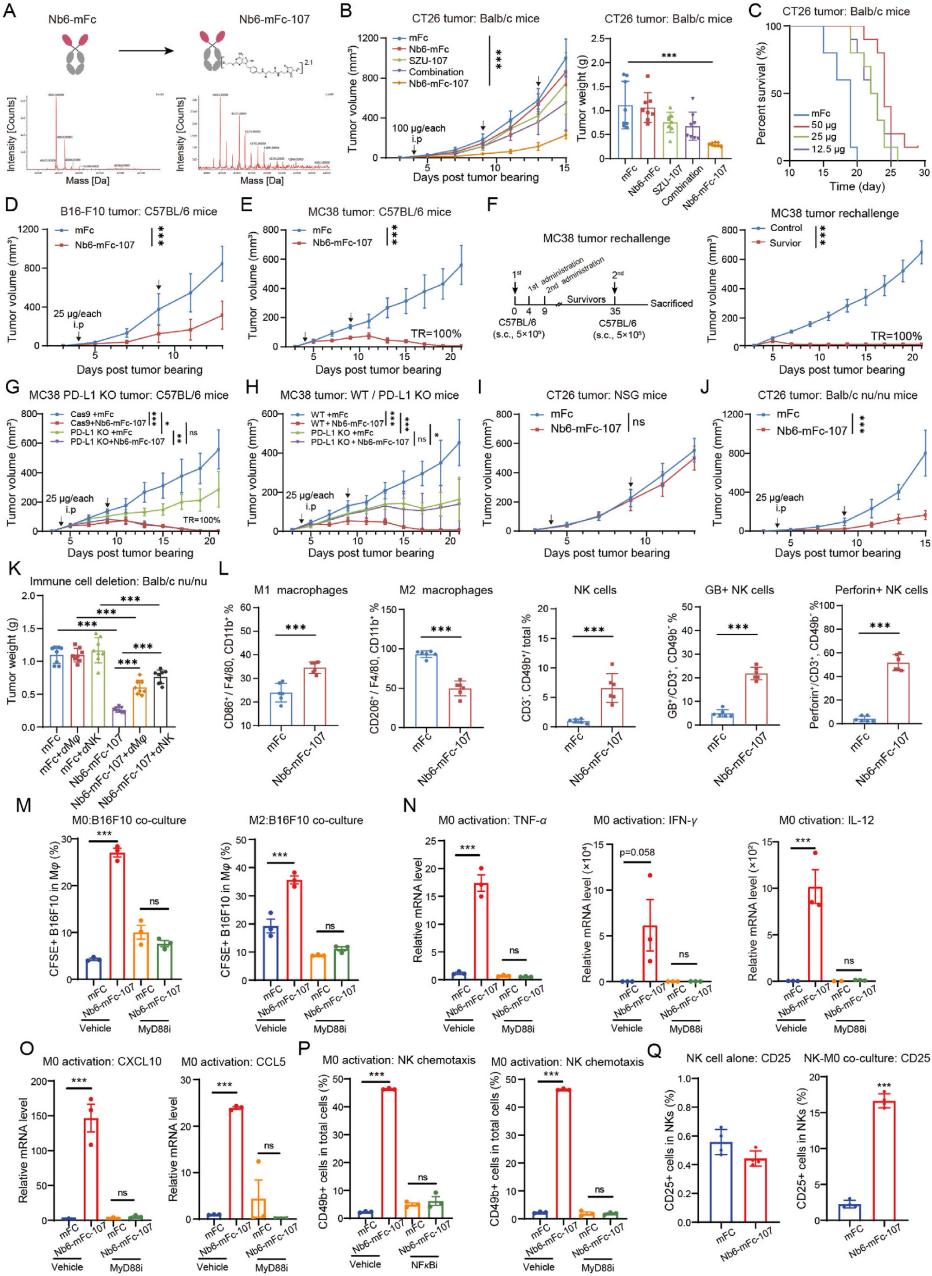
Non-blocking anti-PD-L1 nanobody conjugated to TLR7 agonist mediates macrophage/NK cell-associated antitumor effects. (A) Preparation and characterization of Nb6-mFc-107. The DAR value was calculated mainly based on the increase in molecular weight of Nb6-mFc-107 over the uncoupled Nb6-mFc. (B) Tumor growth kinetics and weights of CT26 cells in mice treated with mFc, Nb6-mFc, SZU-107, combination or Nb6-mFc-107 (*n* = 8). (C) The Kaplan–Meier plot of survival shows the effect of different doses of Nb6-mFc-107 on CT26 tumor bearing mice (*n* = 10). (D) Tumor growth kinetics of B16–F10 cells in mice treated with mFc or Nb6-mFc-107 (*n* = 8). (E) Tumor growth kinetics of MC38 cells in mice treated with mFc or Nb6-mFc-107 (*n* = 8). (F) Schematic of the rechallenge schedules of mice with regressed MC38 tumors. Tumor volume is shown (*n* = 8) for MC38 rechallenge study. (G) Evaluation of antitumor efficacy of Nb6-mFc-107 in a tumor PD-L1 KO model (*n* = 6). (H) Evaluation of antitumor efficacy of Nb6-mFc-107 in a mouse PD-L1 KO model (*n* = 6). (I, J) Tumor growth kinetics of CT26 cells in NSG or Balb/c^nu/nu^ mice treated with mFc or Nb6-mFc-107 (*n* = 8). (K) Antitumor efficacy evaluation of Nb6-mFc-107 after macrophage or NK cell deletion in Balb/c^nu/nu^ mice. (L) Regulatory function of Nb6-mFc-107 on immune cells in the CT26 tumor microenvironment by flow cytometry (*n* = 6). (M) Effect of Nb6-mFc-107 and MyD88 inhibitor TJ-M2020-5 on the ability of M0 and M2 type macrophages to phagocytose B16F10 cells (*n* = 3). (N) Effects of Nb6-mFc-107 and MyD88 inhibitor on cytokine secretion by M0-type macrophages (*n* = 3). (O) Effects of Nb6-mFc-107 and MyD88 inhibitor on chemokine expression by M0-type macrophages (*n* = 3). (P) Migration rate of spleen NK cells co-cultured with M0-type macrophages with or without treatment of Nb6-mFc-107, MyD88/NF-*κ*B inhibitor (*n* = 3). (Q) Activation analysis of splenic NK cells when cultured alone or co-cultured with M0-type macrophages with or without treatment of Nb6-mFc-107 (*n* = 3). The data are presented as mean ± SEM. ∗*P* < 0.05; ∗∗*P* < 0.01; ∗∗∗*P* < 0.001; ns not significant by unpaired *t*-test or one-way/two-way ANOVA followed by Tukey's multiple comparisons test.

Based on these results, we hypothesized that non-blocking Nb6 could be coupled with TLR7 agonists for the preparation of ISAC with antitumor activity. We ligated compound SZU-101 with polyethylene glycol to obtain the intermediate SZU-107 with better solubility and the same activity. Next, SZU-107 was coupled to Nb6-mFc *via N*-hydroxy succinimide (NHS). The purity and PD-L1 binding activity of the ISAC, Nb6-mFc-107, were determined to be unaffected (Supporting Information Fig. S5A and S5B). By mass spectrometry, the drug to antibody ratio (DAR) value of Nb6-mFc-107 was identified as 2.1 (Fig. 2A) and its batch and storage stability was good (Fig. S5C and S5D).

Subsequently, we systematically evaluated the antitumor effects and mechanisms of Nb6-mFc-107. CT26 tumor-bearing mice were administered with 100 μg Nb6-mFc-107 for three doses. Tumor growth kinetics and weights showed that Nb6-mFc-107 significantly inhibited tumor progression (Fig. 2B). The tumor inhibition rates of Nb6-mFc, SZU-101, combination, and Nb6-mFc-107 groups were 4.42%, 32.06%, 39.33%, and 73.60%, respectively. Further, we administered 50, 25, and 12.5 μg/each of Nb6-mFc-107 to CT26 tumor-bearing mice. The survival curves indicated that the efficacy of Nb6-mFc-107 had a slight dose-dependent effect and considerably inhibited tumor growth at lower dosages (Fig. 2C). The efficacy of Nb6-mFc-107 has also been evaluated in other tumor models. B16–F10 is a “cold” tumor model with poor immune cell infiltration. B16–F10 tumor-bearing mice were administered with 25 μg Nb6-mFc-107 for two doses. The IR of Nb6-mFc-107 against B16–F10 was 60.41% (Fig. 2D). MC38 is a highly immunogenic “hot” tumor model. Administration of Nb6-mFc-107 resulted in complete regression of MC38 tumors in 100% of mice (Fig. 2E). Further, we rechallenged MC38 tumors on cured mice, and the tumors again regressed 100%, showing that Nb6-mFc-107 promoted anti-tumor immune memory (Fig. 2F). The aforementioned studies indicate that our ISAC exhibits significant antitumor activity across various tumor types.

## 3 Non-blocking anti-PD-L1 nanobody conjugated to TLR7 agonist mediates macrophage/NK cell-associated antitumor effects

Notably, Nb6 alone did not possess antitumor activity, suggesting that the efficacy of this ISAC is dependent on the delivery of the TLR7 agonist by the anti-PD-L1 antibody. Anti-PD-L1 antibodies are unnecessary for inhibiting activity in PD-L1/TLR7-targeted ISAC. Thus, we aimed to investigate the reliance of the efficacy of Nb6-mFc-107 on the PD-L1 expression in tumor or host cells. In the PD-L1 knockout (KO) MC38 tumor model, Nb6-mFc-107 administration still achieved complete tumor regression (Fig. 2G). However, the inhibitory effect of Nb6-mFc-107 on MC38 tumors was entirely abolished in PD-L1 KO mice (Fig. 2H). The findings indicate that the effectiveness of Nb6-mFc-107 relies on PD-L1 in host cells rather than in tumor cells.

Consequently, we set out to determine which kinds of host immune cells that contribute to the efficacy of Nb6-mFc-107. First, Nb6-mFc-107 failed to inhibit CT26 tumor progression in server combined immune-deficiency NSG mice (Fig. 2I). Next, in T cell-deficient Balb/c^nu/nu^ mice, the IR of Nb6-mFc-107 against CT26 tumors was 78.48%, suggesting that T cells are not necessary (Fig. 2J). Immune cells such as macrophages and natural killer (NK) cells were still present in Balb/c^nu/nu^ mice. Further, we deleted macrophages and NK cells in BALB/c^nu/nu^ mice and found that the efficacy of Nb6-mFc-107 was significantly impaired (Fig. 2K). *In vitro*, Nb6-mFc-107 did not have direct cytotoxic effects on tumor cells and macrophages (Supporting Information Fig. S6). These results suggest that macrophages and NK cells are pharmacological target cells for Nb6-mFc-107. This also coincides with the previously discovered biased targeting of Nb6 to macrophages. Furthermore, we analyzed the immunophenotyping of macrophages and NK cells in the CT26 tumor microenvironment after Nb6-mFc-107 treatment. Flow cytometry results demonstrated that Nb6-mFc-107 promoted intratumoral infiltration of M1-type macrophages and decreased M2-type macrophages (Fig. 2L). Nb6-mFc-107 promoted intratumoral infiltration of NK cells, and it promoted expression of perforin and granzyme B by NK cells (Fig. 2L). Similar results were also observed in the B16–F10 tumor microenvironment (Supporting Information Fig. S7). In addition, considering that dendritic cells (DCs) are also common target cells for TLR7 agonists, we analyzed the effect of Nb6-mFc-107 on DCs. In the CT26 tumors, the results showed that Nb6-mFc-107 administration slightly promoted intratumoral DCs infiltration and activation (Supporting Information Fig. S8A). We then investigated the PD-L1 expression levels of tumor cells, DCs, and macrophages in CT26 and MC38 tumors and discovered that macrophage cells expressed the most PD-L1, whereas DCs expressed just slightly more PD-L1 than tumor cells (Fig. S8B and S8C). This shows that the anti-PD-L1 nanobody Nb6 may preferentially deliver SZU-107 to macrophages, which could also explain the lesser effect on DC cells.

To verify the regulatory effect of Nb6-mFc-107 on intratumoral macrophages, we tested the repolarizing effect of Nb6-mFc-107 on macrophages *in vitro*. In PM and bone marrow-derived macrophage (BMDM) models, Nb6-mFc-107 facilitated macrophage differentiation towards the M1 phenotype and impeded differentiation towards the M2 phenotype (Supporting Information Fig. S9). Further, in a macrophage/tumor cell co-culture model, we found that Nb6-mFc-107 significantly promoted phagocytosis of B16F10 cells by M0-type or M2-type macrophages (Fig. 2M and Supporting Information Fig. S10). Nb6-mFc-107 also promoted the secretion of the anti-tumor factors IFN-*γ* and TNF-*α* by macrophages (Fig. 2N). Notably, these effects were all dependent on the classical MyD88-NF-*κ*B pathway of TLR7 (Fig. 2M and N, and Supporting Information Figs. S10 and S11). In addition, we found that Nb6-mFc-107 increased macrophage secretion of the NK cell activators IL-12, as well as the NK cell chemokines CXCL10 and CCL5 (Fig. 2N and O, Figs. S10 and S11, Supporting Information Table S1). In Transwell assays, we found that macrophages in the lower compartment activated by Nb6-mFc-107 significantly recruited NK cells in the upper compartment, and that this effect was also dependent on MyD88-NF-*κ*B signaling (Fig. 2P). When NK cells were cultured alone, Nb6-mFc-107 did not activate them (Fig. 2Q). However, when NK cells and macrophages were co-cultured, Nb6-mFc-107 significantly promoted NK cell activation (Fig. 2Q). These results suggest that Nb6-mFc-107 exerts its efficacy by orchestrating macrophage and NK cell dependent antitumor immunity.

In summary, we developed a novel ISAC that is a non-blocking anti-PD-L1 nanobody conjugated to TLR7 agonist. We systematically investigated the combination and respective roles of anti-PD-L1 antibody and TLR7 agonist in ISAC. Non-blocking PD-L1 nanobody Nb6 primarily plays a TLR7 agonist delivery role. And non-blocking antibodies may circumvent the possible risk of toxicity associated with blocking activity and allow ISAC drugs to be designed with a broader range of TAA targets. Interestingly, the efficacy of this ISAC is not dependent on tumor cell PD-L1 and T cells, but rather achieves potent anti-tumor efficacy through the modulation of macrophage/NK cell-associated antitumor effects. This PD-L1/TLR7-targeted ISAC demonstrates considerable clinical development potential and may inspire the optimization of other ISAC therapeutics.

## Author contributions

Chao Hu, Xiaolu Yu, Yiru Long and Likun Gong designed the experiments and analyzed the data. Chao Hu and Chen Chen performed the experiments. Chao Hu and Yiru Long prepared the manuscript. Xiaolu Yu, Zhiying Li, Qi Sun and Feng Tang assisted in performing the experiments and preparing the manuscript. All authors approved the final draft of the manuscript.

## Conflicts of interest

The authors declare that they have no competing interests.

## Supporting information

The following is the Supporting Information to this article.
