# Supplementary material for: Non-blocking anti-PD-L1 nanobody conjugated to TLR7 agonist mediates macrophage/NK cell-associated antitumor effects
Source: Acta Pharm Sin B. 2025 May 16;15(7):3819–23. doi: 10.1016/j.apsb.2025.05.005 (PMC12278401; doi:10.1016/j.apsb.2025.05.005)
Supplement: Multimedia component 1 [file mmc1.pdf]

Supporting Information for

Letter to the editor

Non-blocking anti-PD-L1 nanobody conjugated to TLR7 agonist mediates macrophage/NK cell-associated antitumor effects

Chao Hu[a,†], Chen Chen[a,b,†], Xiaolu Yu[a,b,†], Zhiying Li[a,c], Feng Tang[a,b], Qi Sun[a,d], Yiru Long[a,b,*], Likun Gong[a,b,e,*]

[a]*State Key Laboratory of Drug Research, Shanghai Institute of Materia Medica, Chinese Academy of Sciences, Shanghai 201203, China*

[b]*University of Chinese Academy of Sciences, Beijing 100049, China*

[c]*School of Public Health, China Medical University, Shenyang 110122, China*

[d]*School of Chinese Materia Medica, Nanjing University of Chinese Medicine, Nanjing 210023, China*

[e]*Zhongshan Institute for Drug Discovery, Shanghai Institute of Materia Medica, Chinese Academy of Sciences, Zhongshan 528400, China*

Received 15 November 2024; received in revised form 13 March 2025; accepted 6 May 2025

[*]Corresponding authors.

E-mail addresses: s18-longyiru@simm.ac.cn (Yiru Long), lkgong@simm.ac.cn (Likun Gong)

[†]These authors made equal contributions to this work.

1. Materials and methods

1.1. Cell lines

HEK293F cells were purchased from Thermo Fisher and grown in FreeStyle 293 expression medium (12338018, Thermo Fisher). CT26, B16-F10, Raw264.7, HEK293T and MC38 cell lines were purchased from Procell. HEK293T, Raw264.7 and B16-F10 cells were cultured in Dulbecco's modified Eagle's medium (DMEM; MA0212, Meilunbio) supplemented with 1% penicillin/streptomycin (P/S; 15140122, Invitrogen) and 10% heat-inactivated fetal bovine serum (FBS; 10100147, Life Technologies). CT26 and MC38 were cultured in RPMI1640 medium (MA0215, Meilunbio) containing 1% P/S and 10% FBS. All cells were cultured at 37 ℃ in a 5% CO_2 humidified atmosphere.

1.2. Mice

Female (six- to eight-week-old) BALB/c mice (Shanghai Sipp-BK), C57BL/6J mice (Shanghai Sipp-BK), Balb/c$^{nu/nu}$ mice (Vital River Laboratory), NSG mice (Jackson Laboratory), and PD-L1-deficient C57BL/6J mice (Shanghai Model Organisms Center) were maintained under specific pathogen-free (SPF) conditions in the animal facility of the Shanghai Institute of Materia Medica, Chinese Academy of Sciences (SIMM). Animal care and experiments were performed in accordance with SIMM using protocols approved by the Institutional Laboratory Animal Care and Use Committee (IACUC).

1.3. Construction of stable transfected cell lines

The HEK293T-mPD-L1 cell line was generated by a lentiviral system. Briefly, the full murine PD-L1 gene was subcloned into the pLVX-puro vector (Clontech), which was then transfected into HEK293T cells with the package plasmids (pSPAX2 and pMD2.G). Then, the virosomes were collected and transfected into HEK293T cells. After transfection, the transfected HEK293T cells were selected by puromycin and tested by flow cytometry. The PD-L1 KO MC38 cells were also constructed through lentivirus encoding sgRNA (5′-GTATGGCAGCAACGTCACGA-3′). The specific methods are the same as those described in our previous work[1,2].

1.4. Acquisition of PD-L1 nanobodies

The nanobodies Nb6 and Nb16 were obtained in the same way as in our previous study[1], including camel immunization, nanobody library construction and screening of nanobodies by phage display technology.

1.5. Generation and purification of nanobodies

The VHHs of Nb6 and Nb16 were inserted into pFUSE-mIgG2b-Fc vector respectively. The plasmids of VHH-pFUSE-mIgG2b-Fc were transfected into FreeStyle HEK293F cells to express the nanobodies. The SDS-PAGE was used to characterize the nanobodies (15 kDa) with an Fc tag (27 kDa).

1.6. Affinity, endocytosis and targeting of Nb6

For the affinity assay, serial dilutions of Nb6-mFc solution were incubated with HEK293T-mPD-L1 cells (3×10^5 cells/each) for 30 min at 4 ℃, respectively. And then, the cells were incubated with anti-mouse IgG2B (PE; IC0041P, R&D) for detection by flow cytometry.

For the blocking activity, serially diluted Nb6-mFc and Nb16-mFc solutions were mixed with mouse PD-1-hFc protein solution (3 μg/mL), respectively. The mixed solutions were incubated with HEK293T-mPD-L1 cells (3×10^5) for 30 min at 4 ℃. And then, the cells were incubated with Goat anti-human IgG Fc (FITC; ab97264, Abcam) for detection by flow cytometry.

For antibody endocytosis, Nb6-mFc antibody solution (5 μg/mL) was incubated with the target cells (CT26, B16-F10, Raw264.7, PM) (3×10^5 cells/each) for 30 min at 4 ℃, subsequently. The samples were placed for 4 h at 37 or 4 ℃ after replacing the solution with PBS. And then, the cells were incubated with anti-mouse IgG2B (PE) for detection by flow cytometry.

For targeting, CT26 tumor tissues from mice were separated and processed into single cell suspensions, and Nb6-mFc solution (2 μg/mL) was incubated with single cell suspension for 30 min at 4 ℃. The cells were then incubated with anti-mouse IgG2B (PE), Mouse Anti-Mouse CD45.2 (APC-Cy7; 560694, BD Biosciences), ANTI-MO F4/80 AG BM8 (PE-Cy7; 25-4801-82, eBioscience), and anti-mouse/human CD11b (BV421; 101235, BioLegend) for detection by flow cytometry.

1.7. Separation and polarization induction of PM and BMDM in vitro

For peritoneal macrophages (PM), after 3 days of induction with Brewer thioglycolate medium (LA4590, Solarbio), peritoneal macrophages were obtained from mice by peritoneal lavage. Macrophage polarization was treated with 20 ng/mL IFN-γ (Z02916, GenScript) and 100 ng/mL LPS (L6529, Merck) for M1 type polarization or 40 ng/mL IL-4 (HY-P7080, MCE) for M2 type polarization.

For bone marrow-derived macrophages (BMDM), BALB/c mice were sacrificed, and bone marrow cells were collected from tibial. Bone marrow cells were cultured with DMEM medium containing 20 ng/mL M-CSF (Z03275, GenScript) for 4 days. Macrophage polarization was treated as described above.

1.8. PD-L1 induction assays for macrophages

In vitro, PM and Raw264.7 cells were added to 24-well plates (1×10^6 cells/mL, 0.5 mL) and stimulated with 50 mmol/L of SZU-101 for 24 or 48 h. Cell samples were collected and then incubated with Rat Anti-Mouse CD274 (BV650; 740614, BD Biosciences) for detection by flow cytometry.

In vivo, in CT26 tumor bearing BALB/c mice, peritumoral injection of SZU-101 (1 mg/mL, 0.1 mL/each) was performed on Days 5, 7 and 9 after tumor bearing. Tumor tissues were isolated and processed into single cell suspensions on Day 11. And then, cells were incubated with Mouse Anti-Mouse CD45.2 (APC-Cy7), ANTI-MO F4/80 AG BM8 (PE-Cy7), anti-mouse/human CD11b (BV421), Rat Anti-Mouse CD274 (BV650) for detection by flow cytometry.

1.9. Preparation of nanobody-drug conjugate

SZU-101 was connected to PEG to yield the intermediate compound SZU-107. 40 mg of SZU-107, 35 mg of HBTU, 21.34 mg of NHS, and 22 μL of DIPEA were added into 0.5 mL of DMF, and the reaction mixture was stirred at RT. After 30 min, the HPLC indicated the completion. The solution was subjected to preparative HPLC and the fractions containing the products were collected and lyophilized to give a white powder as SZU-107-NHS. Subsequently, the activated ester was dissolved with DMSO. Antibodies and small molecules were reacted in a molar ratio of 1:10. Small molecules were added to Nb6 solution and the reaction was stirred at 4 ℃ for 4 h. After the reaction, the small molecules were removed by filtration with a 10 kDa biofilter membrane and the novel compound Nb6-mFc-SZU-107 was obtained. To identify NDC, the antibodies were denatured to open the disulfide bond, after which the sample was identified by an Xevo G2-XS QTOF mass spectrometer (Waters). The DAR value was calculated mainly based on the increase in molecular weight of Nb6-mFc-SZU-107 over the uncoupled antibody. The stability evaluation of Nb6-mFc-107 was determined by using the above method with different coupling batches of samples and samples left at 4 ℃ for one week, respectively.

1.10. Repolarization of macrophages by Nb6-mFc-107 in vitro

After inducing the polarization of macrophages (PM and BMDM) into M1 type or M2 type respectively, M2-type macrophages were stimulated with Nb6-mFc-107 (5 μg/mL)

and control sample (Nb6-mFc, SZU-101, Combination) at the same dose for 48 h. M1 and M2 markers in macrophages were identified by flow cytometry.

1.11. In vivo tumor models

CT26 (5×10^5), MC38-Cas9 (5×10^5), MC38-PD-L1 KO (5×10^5) or B16-F10 (5×10^5) cells were suspended in PBS and injected subcutaneously into the lateral abdomen of mice respectively. Tumors were measured every 2 days with calipers. Mice were randomly grouped when the average tumor volume reached 40–60 mm^3. Intraperitoneal drug administration was performed on Days 4 and 9 after tumor bearing, respectively. In all model, tumor volume was calculated as Eq. (1):

$$\text{Tumor volume } (mm^3) = \text{Tumor length} \times \text{Width} \times \text{Width} / 2 \qquad (1)$$

And tumor growth curves were plotted. At the end of the experiment, the mice were sacrificed to collect the tumor tissues and major organs for further assay.

1.12. Immunophenotype analysis

Tumor tissues were isolated from mice, then digested with collagenase type IV (1 mg/mL; 40510ES60, Yeasen) and hyaluronidase (1 mg/mL; 20426ES60, Yeasen) solution and filtered through 75-micron nylon mesh (7061011, Dakewe) to prepare single cell suspension. The cells were blocked with anti-CD16/CD32 antibody (553141, BD Biosciences) and stained with indicated surface antibodies. Intracellular antibodies were stained after permeabilization with Cytofix/Cytoperm buffer (554714, BD Biosciences). Flow cytometry analysis was performed using an ACEA NovoCyte, and data processing was performed using NovoExpress software.

1.13. Immune cell deletion model

For macrophage deletion, Balb/$c^{nu/nu}$ mice were treated by intraperitoneal injection of Clodronate liposomes (5 mg/mL, 100 μL/each; 40337ES08, Yeasen) at 2 days before tumor bearing, and Days 4 and 8 after tumor bearing, respectively.

For NKs deletion, Balb/$c^{nu/nu}$ mice were treated by intraperitoneal injection of anti-Asialo-GM1 Antibody (20 μL/each; 146002, Biolegend) at 2 days before tumor bearing, and Days 4 and 8 after tumor bearing, respectively.

1.14. Macrophage phagocytosis assay

M0-type or M2-type BMDM cells (4×10^5 cells/mL) were treated with 5 μg/mL of Nb6-mFc-107, 2 μmol/L of NF-*κ*B inhibitor BAY-11-7082 (HY-13453, MCE), and 30 μmol/L of MyD88 inhibitor TJ-M2020-5 (HY-139397, MCE), respectively, for 24 h. Subsequently, the cell culture supernatant was aspirated, and the CFSE (MB2308, MeilunBio) labeled B16F10 tumor cell (2×10^5 cells/mL) suspension was added, and incubation was continued for 24 h. Cells were collected and the percentage of CFSE$^+$ under the CD11b$^+$ (Brilliant Violet 421 anti-mouse/human CD11b, 101235, BioLegend) gate was detected by flow cytometry.

1.15. Analysis of macrophage cytokines and chemokines

M0-type BMDM cells (4×10^5 cells/mL) were treated with 5 μg/mL of Nb6-mFc-107, 2 μmol/L of NF-*κ*B inhibitor BAY-11-7082, and 30 μmol/L of MyD88 inhibitor TJ-M2020-5, respectively, for 24 h. Total RNA of cells was isolated with TRIzol (10606ES60, Yeasen) and cDNA was generated with an RT reagent kit (RK20428, ABclonal). Quantitative PCR was carried out with 2× SYBR Green Fast qPCR Mix (RK21203, ABclonal) to assess target mRNA expression. The primers were obtained from GENEWIZ.

1.16. NK cell chemotaxis assay

Spleen NK cells were isolated by magnetic bead sorting (Mouse NK Cell Isolation Kit, 19855, Stemcell). M0-type BMDM cells (4×10^5 cells/mL; with or without 5 μg/mL of Nb6-mFc-107) were added into the bottom chamber of a transwell device with a PET membrane with a 3 μm pore size (14322, LABSELECT), and the top chamber contained 5×10^5 cells/mL NK cells in 200 μL complete RPMI-1640 medium. After the co-culture for 4 h, the cells in the bottom chamber were collected and the percentage of CD49b+ (Ms CD49b/Pan-NK Cells FITC DX5, 553857, BD Bioscience) cells was detected by flow cytometry.

1.17. NK cell activation assay

For NK cell culture alone, splenic NK cells (1×10^6 cells/mL) were treated with 5 μg/mL of Nb6-mFc-107 for 24 h after which the percentage of CD25+ (Ms CD25 PE 3C7, 553075, BD Bioscience) was detected by flow cytometry. For NK cell-macrophage co-culture, splenic NK cells (1×10^6 cells/mL) and M0-type macrophages (4×10^5 cells/mL)

were co-cultured and treated with 5 μg/mL of Nb6-mFc-107 for 24 h, after which the proportion of CD25+ in CD49b+ cells was detected by flow cytometry.

1.18. CCK8 assay

B16F10 tumor cells (4×10^5 cells/mL), CT26 tumor cells (4×10^5 cells/mL) or Raw264.7 cells (4×10^5 cells/mL) were seeded in 96-well plates. Cells were treated with 0, 2, 5, 10, 20, 40, and 80 μg/mL of Nb6-mFc-107 or Nb6-mFc, respectively. CCK8 (40203ES60, Yeasen) was added at 24 h and optical density (OD) was measured at 450 nm by an automatic microplate reader SpectraMax (Molecular Devices).

1.19. Structure prediction of Nb6/PD-L1 and Nb16/PD-L1 complex

Amino acid sequence of mouse PD-L1 was obtained from the Uniprot database. AlphaFold2 was utilized to predict the structures of two complexes[3]. We used the Alphafold2-multimer algorithm *via* Google Colab (https://colab.research.google.com/github/sokrypton/ColabFold/blob/main/AlphaFold2.ipynb) to make predictions. Structural presentation of protein complexes and analysis of interaction sites were performed by Discovery studio 2016.

1.20. Statistical methods and software

Statistical analysis was performed using GraphPad Prism V.8 Software. Statistical differences were analyzed by Student's *t*-test or one-way analysis of variance followed by Tukey's multiple comparisons test. Detailed statistical methods and sample sizes in the experiments are described in each figure legend. All statistical tests were two sided and P values <0.05 were considered to be significant.

2. Supporting table

Table S1 Primer sequences for RT-PCR analysis.

Gene	Primer	Sequence (5′–3′)
Tnf	FW	CCGATGGGTTGTACCTTGTC
	RV	GGCAGAGAGGAGGTTGACTTT
Il12a	FW	AGACATCACACGGGACCAAAC
	RV	CCAGGCAACTCTCGTTCTTGT
Cxcl10	FW	CCAAGTGCTGCCGTCATTTTC
	RV	GGCTCGCAGGGATGATTTCAA
Ifng	FW	CGGCACAGTCATTGAAAGCC
	RV	TGCATCCTTTTTCGCCTTGC

Ccl5	FW	GCTGCTTTGCCTACCTCTCC
	RV	TCGAGTGACAAACACGACTGC

3. Supporting figures

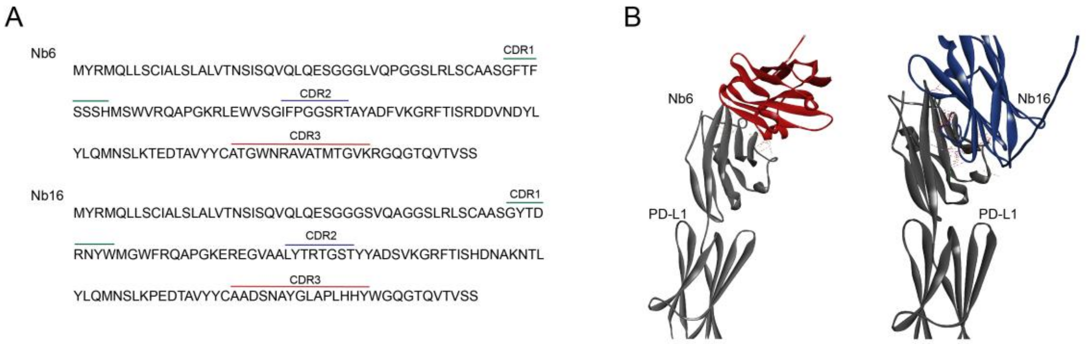

Figure S1 Sequence and structure of the nanobodies. (A) Sequence of nanobodies Nb6 and Nb16. (B) Complex structures of Nb6/PD-L1, Nb16/PD-L1 predicted by AlphaFold.

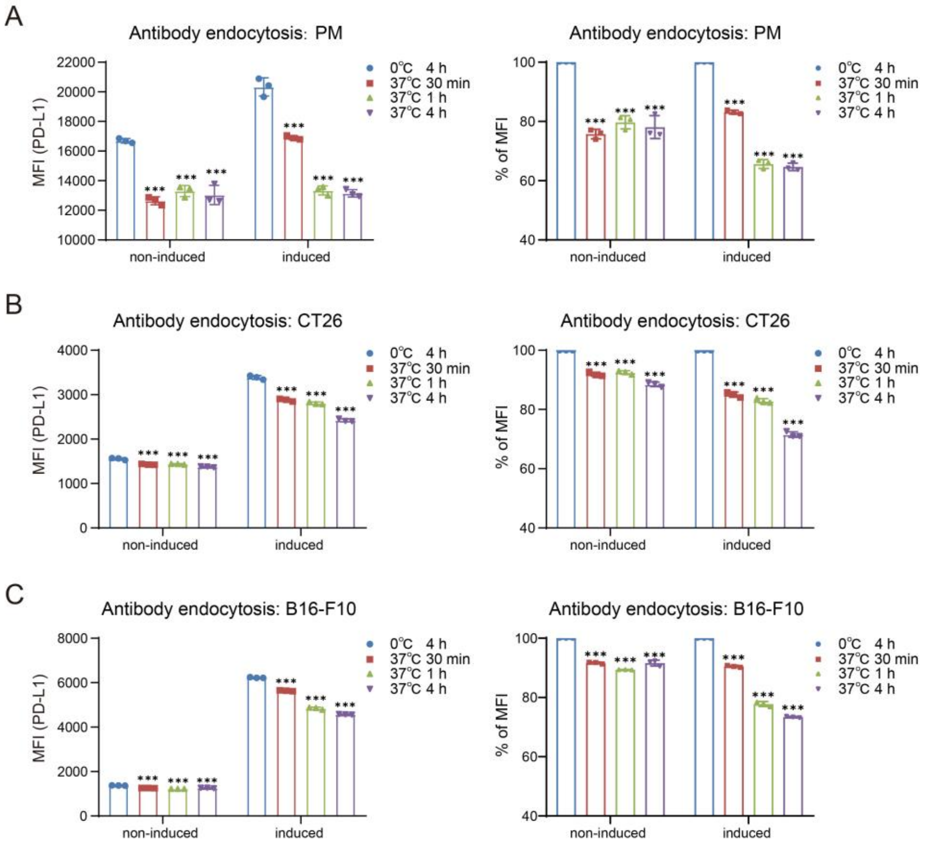

Figure S2 Endocytosis analysis of Nb6-mFc. Endocytosis analysis of Nb6-mFc in PM, CT26, and B16-F10 cells induced by IFN-γ for PD-L1 expression or not (n = 3). The

data are presented as the mean ± SEM. ***P < 0.001 by one-way ANOVA followed by Tukey's multiple comparisons test.

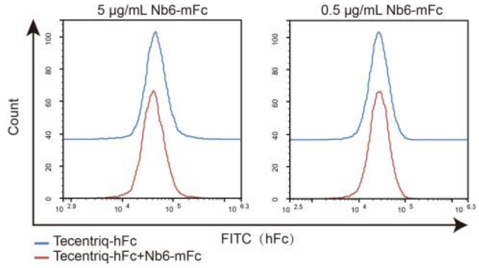

Figure S3 Epitope competition analysis of Nb6-mFc and Tecentriq. Flow cytometry histograms show that Tecentriq (Atezolizumab) does not affect Nb6 binding to cell surface PD-L1.

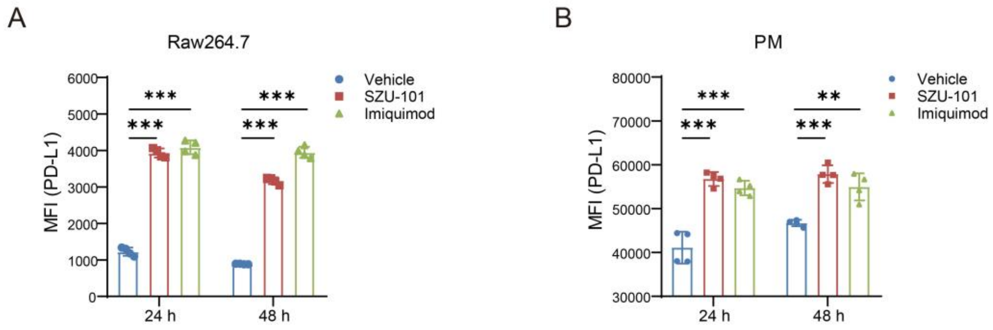

Figure S4 Regulation of PD-L1 by TLR-7 agonists. Changes in PD-L1 in Raw264.7 cells or PM after incubation with 50 mmol/L of SZU-101 and Imiquimod for 24 h and 48 h, respectively (n = 4). The data are presented as the mean ± SEM. **P < 0.001; ***P < 0.001 by one-way ANOVA followed by Tukey's multiple comparisons test.

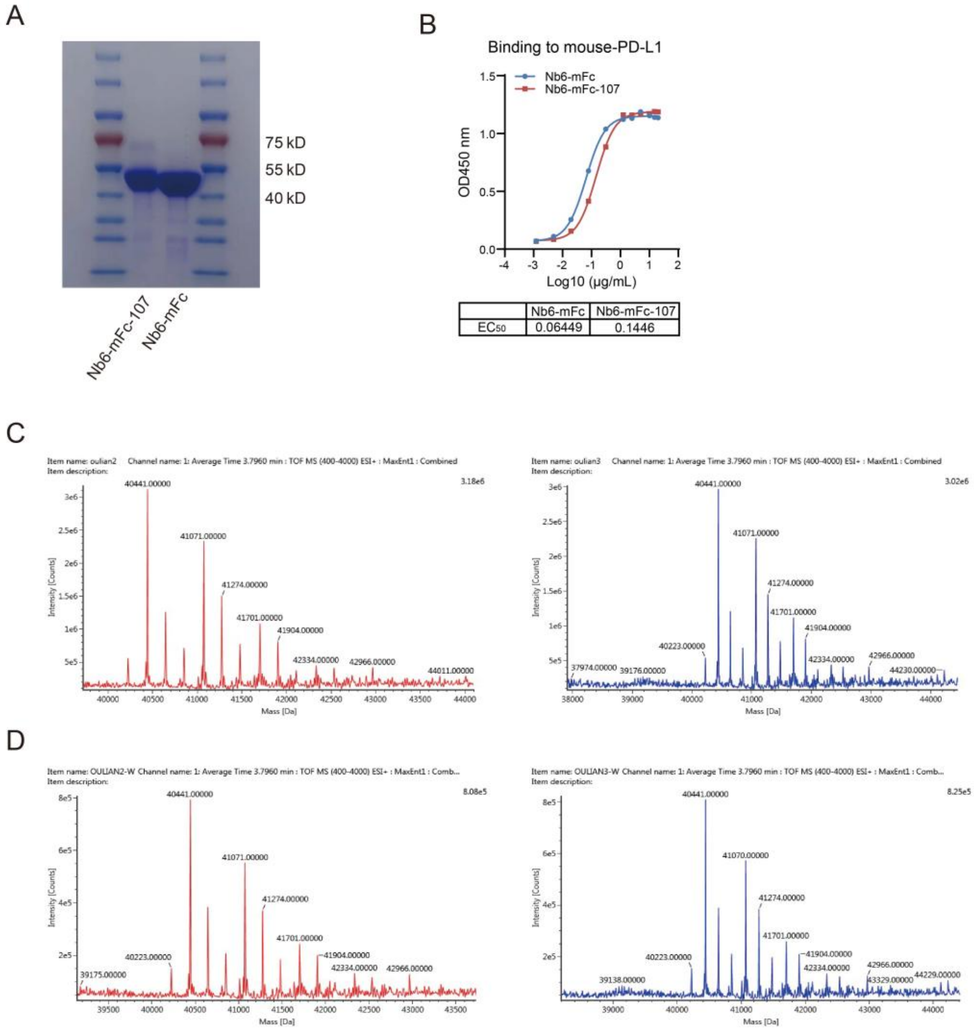

Figure S5 Identification and stability analysis of Nb6-mFc-107. (A) Comparison of molecular weight of samples before and after coupling. (B) Binding activity of Nb6-mFc-107 to PD-L1. (C) Mass spectrometry of Nb6-mFc-107 with different coupling batches. (D) After one week of storage at 4 ℃, mass spectrometry of the Nb6-mFc-101.

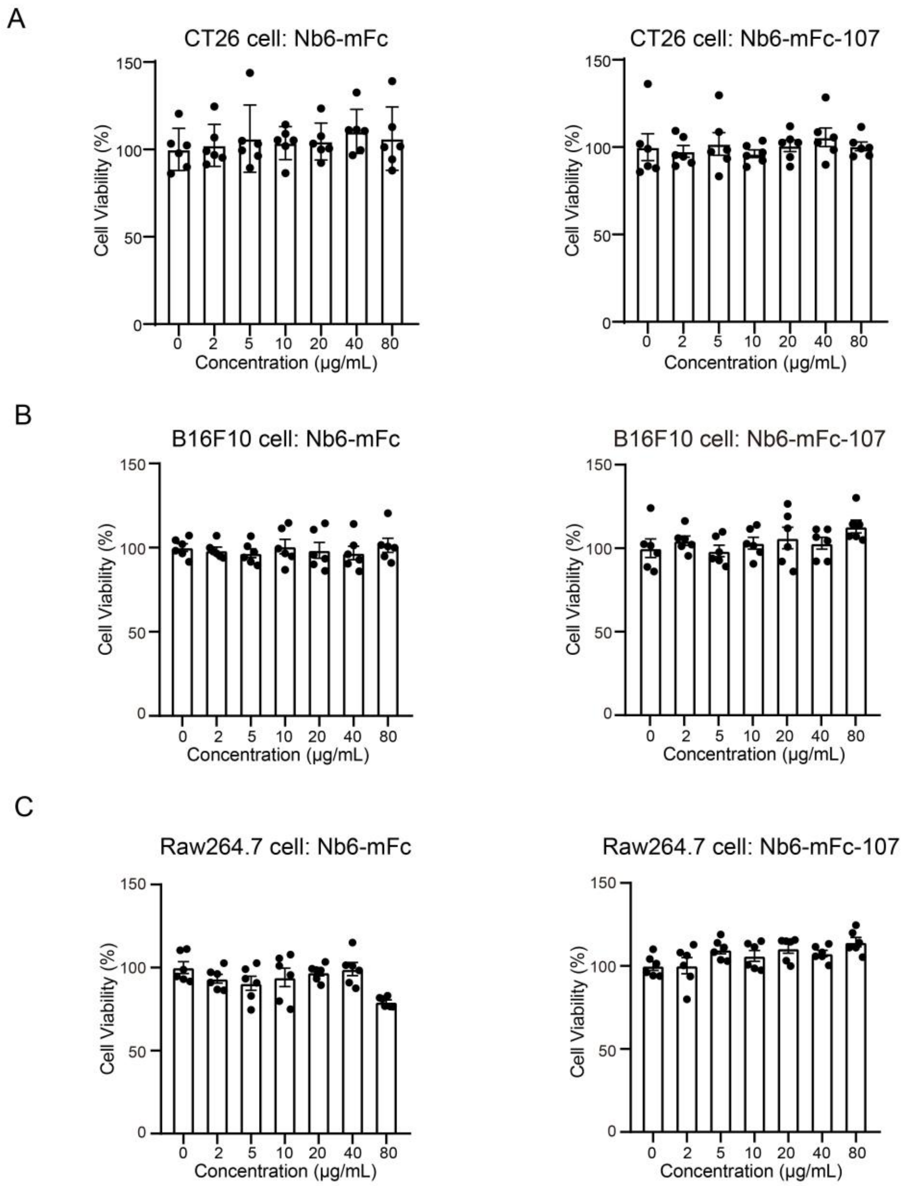

Figure S6 Cytotoxicity assay of Nb6-mFc-107 on tumor cells and macrophages. CT26 cells, B16F10 cells and Raw264.7 cells were treated by serial concentrations of Nb6-mFc or Nb6-mFc-107 administration for 24 h and then cell growth was analyzed by CCK8. (n = 6). The data are presented as the mean ± SEM.

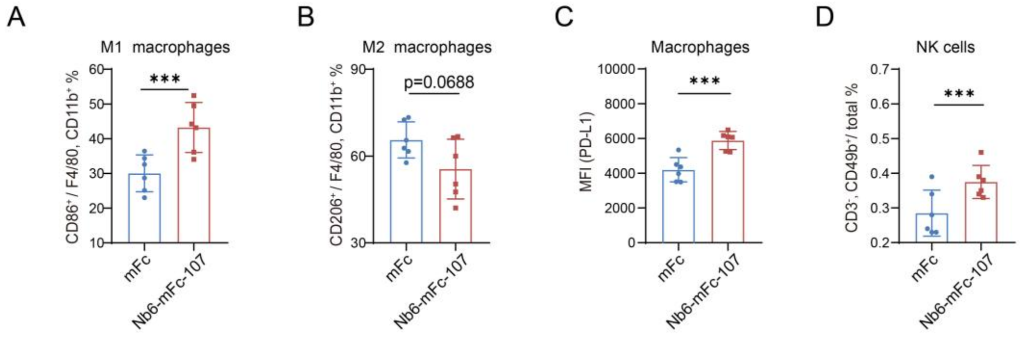

Figure S7 Regulatory function of Nb6-mFc-107 on immune cells in the B16-F10 tumor microenvironment. After Nb6-mFc-107 administration, B16-F10 intra-tumoral macrophages and NK cells were analyzed by flow cytometry (n = 6). The data are presented as the mean ± SEM. ***$P < 0.001$ by unpaired t-test.

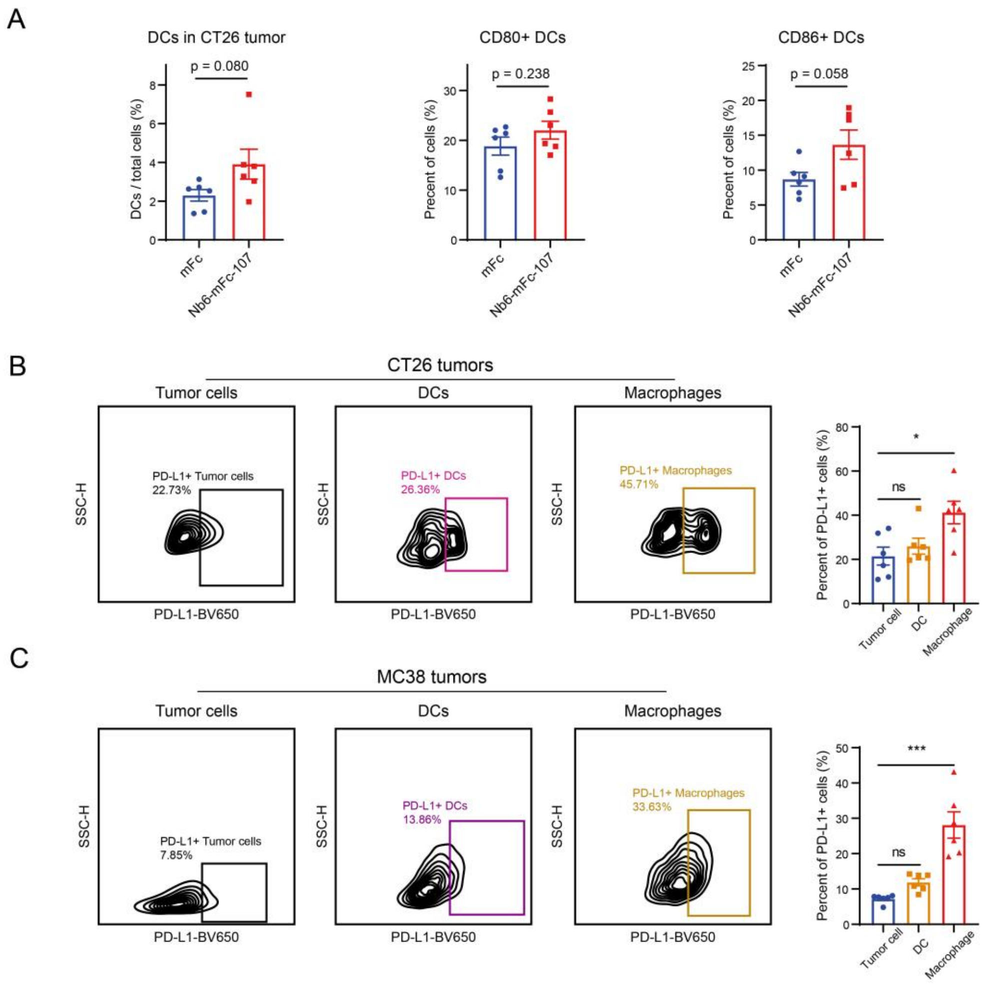

Figure S8 Intratumoral infiltration DCs analysis and PD-L1 level analysis. (A) After Nb6-mFc-107 administration, CT26 intratumoral DCs were analyzed by flow cytometry (n = 6). (B, C) PD-L1 expression analysis of tumor cells, DCs and macrophages within CT26 and MC38 tumors by flow cytometry (n = 6). The data are

presented as the mean ± SEM. ns, not significant; *$P < 0.05$; ***$P < 0.001$ by unpaired *t*-test or one-way ANOVA followed by Tukey's multiple comparisons test.

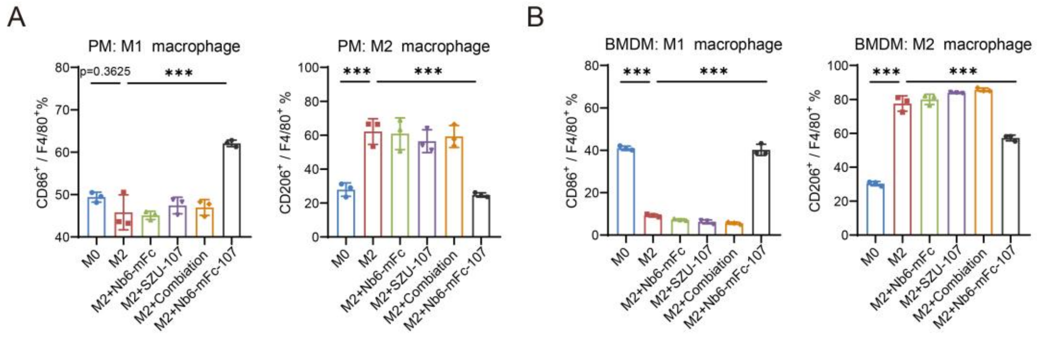

Figure S9 *In vitro* verification of macrophage repolarization function of Nb6-mFc-107. (A) Repolarization of PM by Nb6-mFc-107 ($n = 3$). (B) Repolarization of BMDM by Nb6-mFc-107 ($n = 3$). The data are presented as the mean ± SEM. ***$P < 0.001$ by one-way ANOVA followed by Tukey's multiple comparisons test.

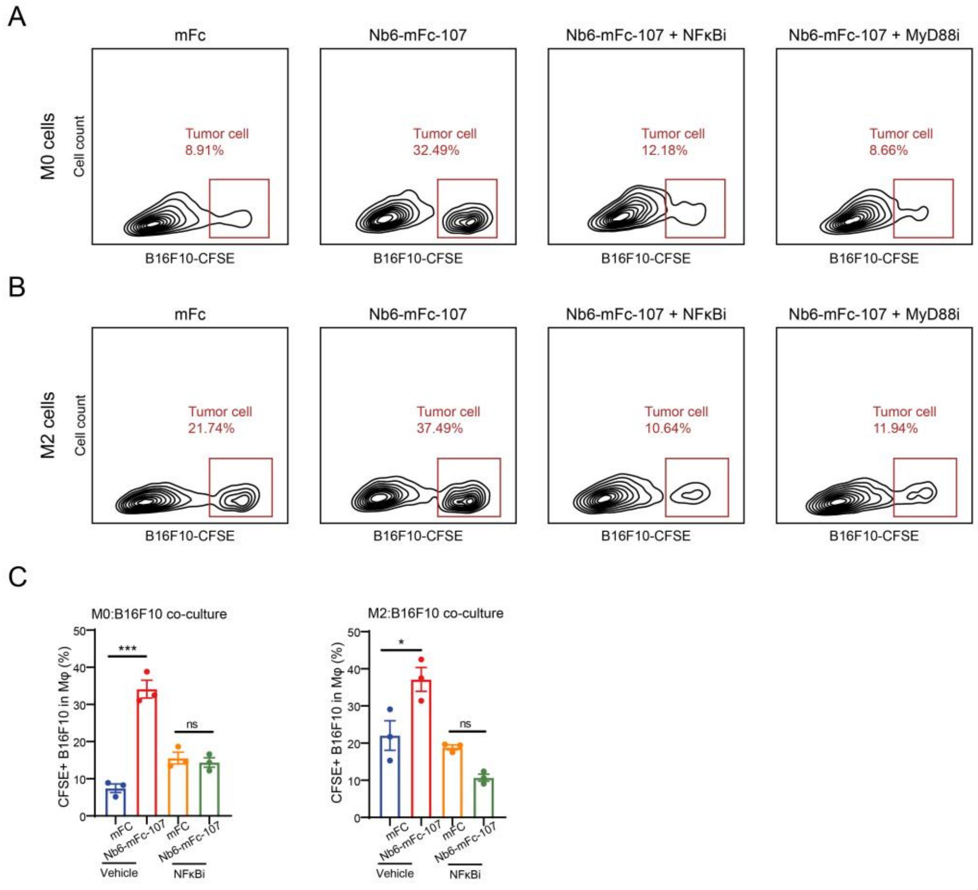

Figure S10 Nb6-mFc-107 promotes phagocytosis of tumor cells by macrophages. (A, B) Representative flow cytometry data for effect of Nb6-mFc-107, MyD88 inhibitor TJ-M2020-5 and NF-*κ*B inhibitor BAY-11-7082 on the ability of M0 and M2 type macrophages to phagocytose B16F10 cells (n = 3). (C) Statistical analysis for effect of Nb6-mFc-107 and NF-*κ*B inhibitor BAY-11-7082 on the ability of M0 and M2 type macrophages to phagocytose B16F10 cells (n = 3). The data are presented as the mean ± SEM. ns, not significant; *P < 0.05; ***P < 0.001 by one-way ANOVA followed by Tukey's multiple comparisons test.

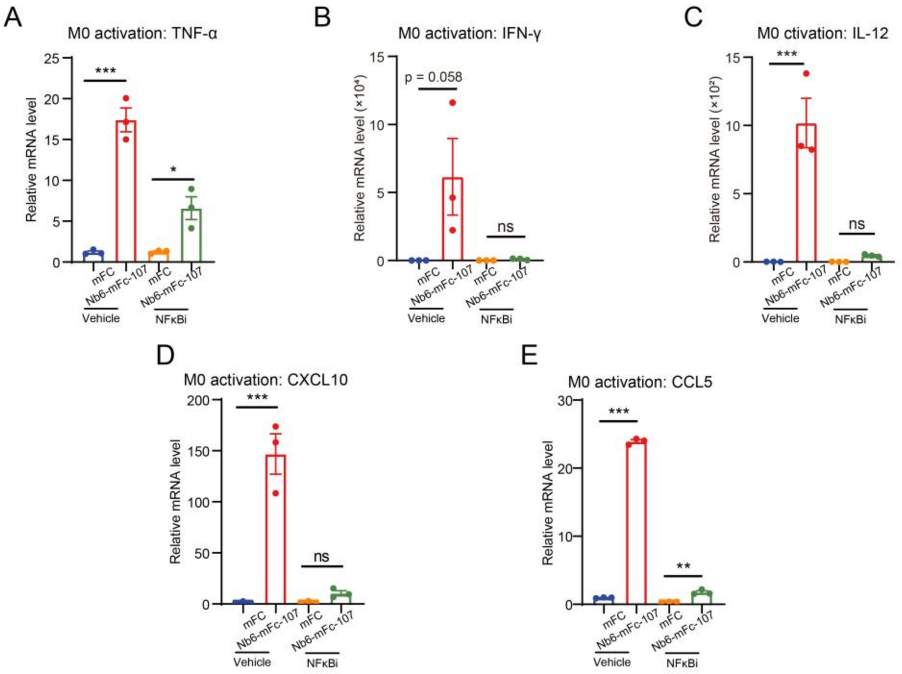

Figure S11 Nb6-mFc-107 promotes the production of anti-tumor-associated cytokines and chemokines by macrophages. (A–F) Effects of Nb6-mFc-107 and NF-*κ*B inhibitor BAY-11-7082 on cytokine and chemokine expression by M0-type macrophages (n = 3). The data are presented as the mean ± SEM. ns, not significant; *P < 0.05; **P < 0.01; ***P < 0.001 by one-way ANOVA followed by Tukey's multiple comparisons test.

References

1 Yu XL, Long YR, Chen BF, Tong YL, Shan MW, Jia XM, et al. PD-L1/TLR7 dual-targeting nanobody-drug conjugate mediates potent tumor regression *via* elevating tumor immunogenicity in a host-expressed PD-L1 bias-dependent way. *J Immunother Cancer* 2022;**10**:e004590.

2 Long YR, Chen RQ, Yu XL, Tong YL, Peng XH, Li FL, et al. Suppression of tumor or host intrinsic CMTM6 drives antitumor cytotoxicity in a PD-L1-independent manner. *Cancer Immunol Res* 2023;**11**:241-60.

3 Mirdita M, Schütze K, Moriwaki Y, Heo L, Ovchinnikov S, Steinegger M, et al. ColabFold: making protein folding accessible to all. *Nat. Methods* 2022;**19**:679-82.
